# Definitions and consequences of current alignment techniques and phenotypes in total knee arthroplasty (TKA) – there is no winner yet

**DOI:** 10.1186/s40634-023-00697-7

**Published:** 2023-11-22

**Authors:** Theofilos Karasavvidis, Cale A. Pagan Moldenhauer, Sébastien Lustig, Jonathan M. Vigdorchik, Michael T. Hirschmann

**Affiliations:** 1 Adult Reconstruction and Joint Replacement Service, Department of Orthopaedic Surgery, Hospital for Special Surgery, 535 East 70th Street, New York, NY 10021 USA; 2 Department of Orthopaedic Surgery and Sports Medicine, Croix-Rousse Hospital, Lyon, 69004 France; 3 Department of Orthopaedic Surgery and Traumatology, Kantonsspital Baselland, Bruderholz, CH-4101 Switzerland; 4 Department of Clinical Research, Research Group Michael T. Hirschmann, Regenerative Medicine & Biomechanics, University of Basel, Basel, CH-4001 Switzerland

**Keywords:** TKA alignment, Knee phenotypes, Personalized arthroplasty

## Abstract

Dissatisfaction following total knee arthroplasty (TKA) has been extensively documented and it was attributed to numerous factors. In recent years, significant focus has been directed towards implant alignment and stability as potential causes and solutions to this issue. Surgeons are now exploring a more personalized approach to TKA, recognizing the importance of thoroughly understanding each individual patient’s anatomy and functional morphology. A more comprehensive preoperative analysis of alignment and knee morphology is essential to address the unresolved questions in knee arthroplasty effectively. The crucial task of determining the most appropriate alignment strategy for each patient arises, given the substantial variability in bone resection resulting from the interplay of phenotype and the alignment strategy chosen. This review aims to comprehensively present the definitions of different alignment techniques in all planes and discuss the consequences dependent on knee phenotypes.

**Level of evidence** V.

## Introduction

Comprehensive data derived from various national joint registries has consistently demonstrated that total knee arthroplasty (TKA) exhibits remarkable long-term implant durability and survival rates in patients experiencing severe knee osteoarthritis [1, 2]. Nonetheless, patient dissatisfaction following an uncomplicated primary TKA is well documented and stands at an average rate of 10% [3, 4].

Efforts to tackle this phenomenon have now focused on implant positioning and limb alignment [5]. Suboptimal alignment of the limb during TKA may result in altered knee kinematics, component wear, and early implant failure necessitating revision TKA procedures [6–8]. An improved understanding and proficient implementation of the ideal alignment techniques have the potential to increase patient satisfaction, optimize functional outcomes, prolong implant longevity, and diminish complications associated with TKA. In recent years, multiple alignment targets have emerged accompanied by a proliferation of surgical techniques [9]. However, for several decades orthopaedic surgeons have universally pursued the goal of a neutral mechanical alignment (MA) as a primary objective [10, 11]. This is rooted in the principle that the postoperative coronal alignment of the lower limbs should ideally fall within a range of ±3° from a neutral mechanical axis [12–14]. The mechanical axis, which passes through the center of the knee, facilitates a balanced mediolateral load distribution, thereby minimizing implant wear and reducing the risk of component loosening [15, 16]. To achieve this, surgeons have traditionally employed systematic approaches for more simplicity and reliability. However, this systematic implant positioning disregards patient-specific knee joint anatomy, as implants are consistently placed in the same manner for every patient, without accounting for the constitutional alignment of each patient, which is the alignment they have had since they reached skeletal maturity [7, 17].

The recently ongoing discussion about personalized alignment was fostered by the knee phenotype concept, which was introduced by Hirschmann et al. [18]. In multiple landmark papers the authors have highlighted the variability of coronal alignment in healthy and even more in osteoarthritic knees [19–23]. Furthermore, not only is the bony alignment variable, but also the joint play, which refers to the laxity of each knee compartment [24]. The authors emphasized the importance of phenotyping the knees before TKA, simulating the consequences of the bone cuts, and resulting necessity of soft tissue balancing procedures with each alignment technique [21, 25–27].

This review aims to comprehensively present the definitions of different alignment techniques in all planes and discuss the consequences dependent on knee phenotypes.

## Alignment definitions

**Fig. 1:**
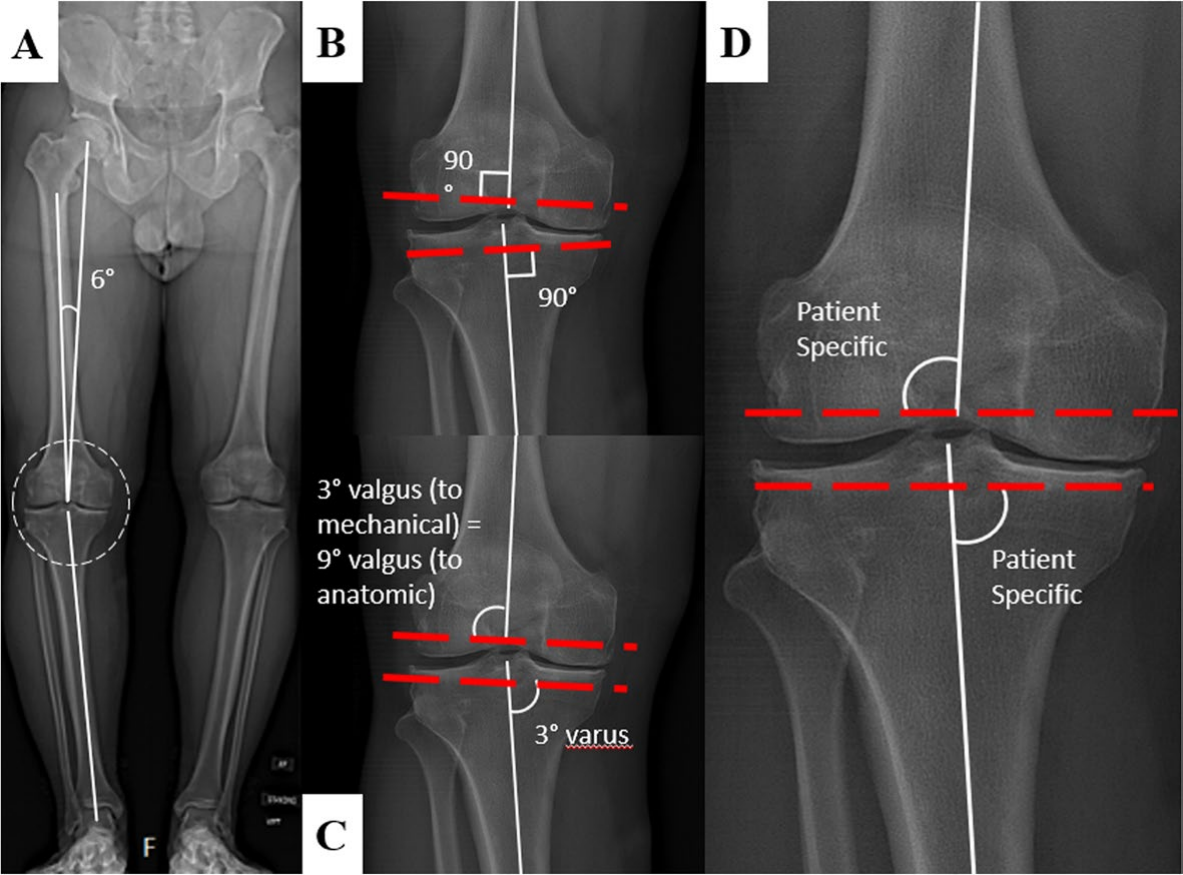
**a** Long-leg radiograph demonstrating the mechanical axis relative to the anatomical axis of the lower extremity. **b** Mechanical alignment of the knee places components at 90° with the mechanical axis of each bone. **c** Anatomical alignment aims for a lateral distal femoral angle (LDFA) of 3° valgus to the femoral mechanical axis (also commonly described as 9° valgus to the femoral anatomical axis). The tibial components are placed with a medial proximal tibial (MPTA) of 3° varus. **d** Individualized alignment techniques. Image reproduced from original work with permission from Dr. Jonathan Vigdorchik

Introduced by John Insall in 1985, the MA technique represents an alignment strategy that seeks to align the femoral and tibial components perpendicular to the mechanical axis of each bone segment, ultimately achieving a neutral hip-knee-ankle angle (HKA) and joint line (Fig. 1) [28]. To achieve balanced mediolateral soft-tissue tension and equalize flexion-extension gaps, surgeons employ either measured resections or gap balancing techniques [29]. MA intentionally disregards individual variations in alignment, morphology, and biomechanics for a high reliability and simplicity. However, this approach comes with potential problems such as lateral column lengthening, distal femoral prosthetic overstuffing, increased patellofemoral retinacular tension, altered native knee kinematics through the arc of knee flexion, and eventually potential technical difficulties in correcting knee imbalance due to neglecting joint line height and obliquity [10, 30]. Even though the MA technique still continues to be the prevailing choice for TKA, additional research is warranted to investigate the accuracy achieved with conventional manual techniques and to establish safe ranges for limb alignment in TKA, taking into account individualized patient anatomy and knee kinematics [10, 31, 32].

A drawback associated with MA pertains to the substantial requirement of soft tissue release, especially in cases of severe deformities, necessitating extensive ligament release to attain balanced gaps [33]. To mitigate the extent of soft tissue releases, the approach of adjusted mechanical alignment (AMA) was introduced [34, 35]. This technique involves performing a tibial cut still perpendicular to the mechanical axis, but the distal femoral cut can be adjusted by up to 3° in either varus or valgus direction, contingent upon the individual extension gap difference [33, 36]. An intentional varus cut in AMA serves as an integral component of the balancing technique, when removal of osteophytes and capsular release fail to achieve a balanced extension gap [37]. This adaptation of the femoral component position offers the advantage of achieving balanced gaps without resorting to excessive releasing techniques, such as pie crusting. However, a noteworthy disadvantage is that the cut of the distal femur may be non-anatomical in certain patients, as some patients have a constitutional valgus of the femur [38, 39]. It is evident that AMA does not truly qualify as a personalized alignment technique, as it fails to consistently restore constitutional alignment in the majority of cases [33, 36].

Initially described by Hungerford and Krackow, the anatomic alignment (AA) technique attains mechanical neutrality by reproducing the oblique joint line characteristic of native knees [40]. This strategy involves distal femur resections in 9° of valgus (relative to the anatomic axis of the femur) and tibial resections in 3° of varus to restore the native angulation of the joint line during extension and achieve a neutral mechanical axis, assuming a 6° difference between the anatomic and mechanical axis. Advocates of AA suggest that it results in better load distribution on the tibial component and improved patellofemoral biomechanics, due to reduced risk of ligament stretching in flexion [41]. However, only the average coronal joint line orientation is in line with AA. Only about 20% of knee phenotypes represent AA [18]. Technical challenges in the 1980s, pertaining to implant design (specifically the Porous-Coated Anatomic prosthesis) and the achievement of accurate bone cuts with the potential for excessive (> 3°) varus alignment of the lower limb, led to limited adoption of AA [42].

Clearly, the distribution of native limb alignment follows a Gaussian pattern, where only 5% to 5.5% of patients exhibit a natural MA [11]. Thus, the pursuit of improved outcomes and restoration of native knee kinematics drove the adoption of more individualized alignment strategies in TKA. The kinematic alignment (KA) technique, introduced by Howell et al. in 2008, aims to replicate the individual’s native limb and joint line while preserving the normal axes of rotation around the knee joint [43]. This approach involves symmetric anatomic cuts on the femur and tibia to compensate for cartilage loss resulting from these resections. KA relies solely on bone cuts to achieve alignment and seeks to maintain the knee’s ligamentous stability and kinematics. Knee balance is accomplished by modifying the tibial cut’s orientation [44]. In KA there are by definition no soft-tissue releases and soft-tissue malalignments are addressed through bone resection, aiming to preserve individual anatomy and potentially enhance functional and clinical outcomes [45, 46]. Even though critics claim that KA may lead to early failure in patients with substantial alignment deformities as it ignores overall limb alignment, in addition to potential patellofemoral maltracking related to internal rotation of the femoral component [47], multiple studies found no significant impact on implant survival or functional scores in short and midterm follow-ups [37, 48–51]. Future studies assessing the safety of KA should clearly describe that no boundaries were applied when restoring native alignment.

Variations of the KA technique emerged due to concerns about the implant longevity when positioned at extremes, leading to the development of the restricted kinematic alignment (rKA) concept. rKA sets specific boundaries to avoid excessive implant positioning in patients with significant limb deformities, as suggested by Vendittolli et al., who proposed ± 3 of neutral for the HKA angle, ± 5 for the medial proximal tibial angle (MPTA), and ± 5 for the lateral distal femoral angle (LDFA) as boundaries [52]. The rKA approach prioritizes the restoration of the femoral component, focusing on joint line obliquity first and subsequently adjusting the tibial component positioning.

In contrast, the inverse kinematic alignment (iKA) technique favors the restoration of tibial joint line obliquity as the initial step, followed by resections on the femur [44]. This process involves the restoration of the native tibial anatomy by precisely removing bone and cartilage from the medial and lateral tibial condyles, matching the thickness of the implant [44, 53]. Following the patient-specific, anatomical tibial cut, the subsequent steps of the procedure remain identical to a conventional gap balancing technique. No soft tissue releases are performed, and the procedure is guided by maintaining the HKA angle within the range of 174° to 183°. The key distinction between KA and iKA lies in their respective methods for achieving knee balance. In KA, the knee balance is attained by altering the orientation of the tibial cut, whereas in iKA knee balance is achieved by modifying the orientation of the femoral cuts [44, 53].

TKA with functional alignment (FA) seeks to position components in a manner that minimally affects the soft-tissue envelope, thereby restoring the plane and obliquity of the joint to align with the natural orientation dictated by the surrounding soft tissues. Preoperatively, surgery is planned to achieve neutral MA or KA, but intraoperatively it combines methods of gap balancing, measured resection, and predictive modeling, utilizing robotic or computer-assisted platforms to virtually position implants and minimize soft-tissue releases, eventually allowing restoration of sagittal knee balance [9]. The functional approach is more about 3D positioning of implants than limited to coronal alignment, since it combines coronal, rotational and sagittal positioning of both femur and tibia component [54, 55].

Despite not being fully optimized, individualized alignment techniques such as KA and FA are demonstrating promising outcomes and have the potential to replace fixed alignment strategies in the near future [37, 45, 56, 57]. However, nowadays there is such a wide array of alignment concepts in TKA that even knee experts may find it challenging to comprehend and distinguish one concept from another (Table 1) [31].

**Table 1:** Overview of different alignment strategies

	**Alignment**	**Tibia cut**	**Soft tissue release**	**Distal femur cut**	**HKA**	**Gaps goals**	**Rotation**
**Tibia first**	**Mechanical**	Always 0°	Osteophytes removal, capsular release, ligament release (pie crusting)	Always 0°	Always 0°	Equal	Always 3° ER
	**Adjusted Mechanical**	Always 0°		Depends on extension gap difference (3° varus-3° valgus)	Between 3° varus and 3° valgus	Equal	Depends on flexion gap difference (up to 9° ER)
	**Constitutional Varus**	Depends on MPTA	Similar to MA	Always 0°	Between 3° varus and 0°	Equal or Flexion > Extension (2mm)	3° less ER compared to AMA
	**Anatomic**	2–3° varus	Over release 2–3 mm	3° of valgus	Always 0°	Equal	3° less ER compared to AMA
	**Inverse Kinematic**	Depends on cartilage loss	No release	Depends on tibia	Between 5° varus and 3° valgus	Lateral flexion gap > medial flexion gap	4–6° less ER compared to AMA
	**Functional**	Depends on cartilage loss	Minimal (dMCL)	Depends on extension gap difference (5° varus-5° valgus)	Between 5° varus and 5° valgus	Lateral flexion gap > medial flexion gap	4–6° less ER compared to AMA
**Femur first**	**Kinematic**	Depends on femur cuts and gap difference	Minimal	Depends on cartilage loss and individual anatomy	Between 5° varus and 5° valgus	Lateral flexion gap > medial flexion gap	Depends on cartilage loss and individual anatomy
	**Restricted Kinematic**	Depends on femur cuts and gap difference	Minimal	Depends on cartilage loss and individual anatomy	Between 3° varus and 3° valgus	Lateral flexion gap > medial flexion gap	Depends on cartilage loss and individual anatomy

Current literature is characterized by a lack of long-term studies investigating the outcomes and limitations of various alignment strategies in patients with different varus, neutral, and valgus phenotypes. Over the last year, simulation studies attempted to pursue these research questions and demonstrated that the extent of bone resection varies significantly depending on both the phenotype and the selected alignment strategy [26, 27]. Those simulations can serve as valuable tools to aid the treating surgeon in identifying the most suitable alignment strategy for each individual patient. For instance, when assessing the most prevalent varus phenotype, Schelker et al. showed that MA would lead to a 6 mm elevation of the tibial medial joint line and a 3 mm lateral distalization of the femoral condyle, AA would result in no change in joint line obliquity but a 3 mm lateral distalization of the femoral condyle, while rKA would cause a 3 mm elevation of the tibial medial joint line and a 3 mm lateral distalization of the femoral condyle. Notably, KA would not result in any change in joint line obliquity [27]. Hence, the limitations of each alignment strategy with each knee phenotype should be a point of emphasis for future studies in the field.

Additionally, there is a critical need for a well-defined approach to transition safely from MA to a more individualized alignment TKA [31, 32]. This entails establishing safe zones for tibial and femoral component positioning, tailored to specific knee phenotypes aiming to optimize outcomes [31, 32]. It should also be noted that current studies have focused on alignment in the coronal plane, with limited data available on the effects of sagittal and axial alignment. To achieve optimal results, alignment should be regarded as a triad encompassing all three planes, allowing for comprehensive bone resections and precise implant positioning [10].

## Knee phenotypes classifications

**Fig. 2:**
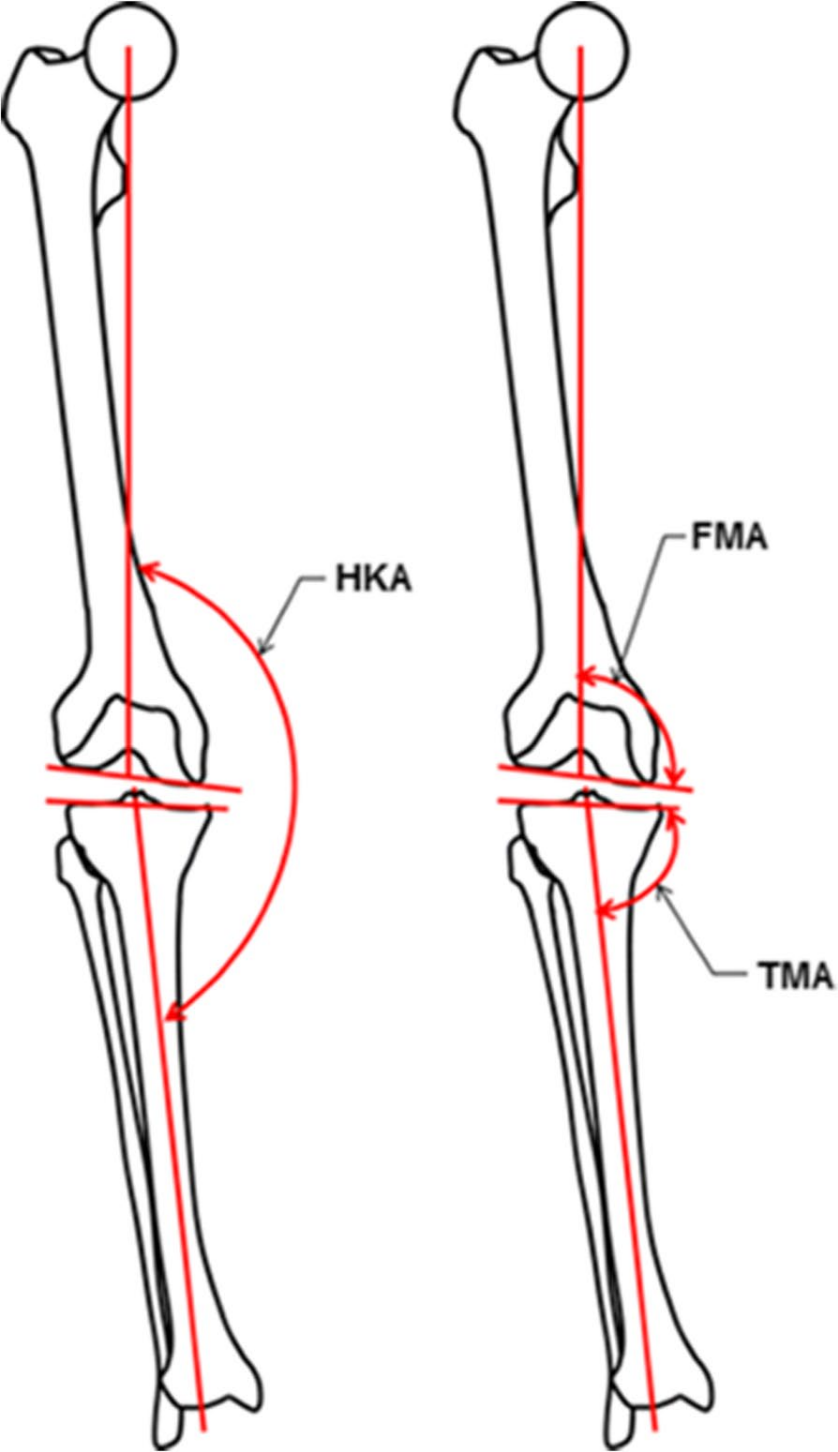
The measured angles: hip–knee–ankle angle (HKA, the angle is formed by the lines connecting the centers of the femoral head, the knee and the talus), femoral mechanical angle (FMA, the angle between the femoral mechanical axis and a tangent to the distal femoral condyles) and tibial mechanical angle (TMA, the angle between the tibial mechanical axis and a tangent to the proximal tibia joint surface). Image reproduced from original work with permission from Prof. Dr. Michael Hirschmann

Bellemans et al. introduced the concept of constitutional varus, demonstrating that based on long leg radiographs a considerable portion of the normal population reaches skeletal maturity with native alignment that deviates from 0 degrees [7]. By understanding the differences of the knees among diverse populations, knee surgeons are able to plan and provide a more personalized approach to TKA [19, 58–60]. The HKA angle, femoral mechanical angle (FMA), tibial mechanical angle (TMA), and joint line convergence angle (JLCA) are valuable measurements in describing coronal alignment (Fig. 2) [61]. Significant variation in overall coronal limb alignment (HKA), femoral (FMA) and tibial coronal alignment (TMA) among osteoarthritic knees is indicated in the literature [19, 20, 60]. The prevalence of MA highlights a lack of awareness concerning the variation of these angles [21].

Various classification systems have been devised to categorize native knee phenotypes based on their coronal alignment characteristics. Lin et al. in 2018 proposed a classification system comprising 27 potential knee phenotypes, of which only 5 were deemed clinically relevant [62]. In 2021 MacDessi et al. proposed the Coronal Plane Alignment of the Knee (CPAK) Classification [63]. This system assesses coronal knee phenotypes based on constitutional limb alignment and joint line obliquity (JLO), that can be determined by calculating the mechanical LDFA and the mechanical MPTA. The constitutional limb alignment, represented as varus, neutral, or valgus, is termed the arithmetic hip-knee-ankle angle (αHKA) and is calculated as MPTA - LDFA. The JLO is described as apex distal, neutral, or apex proximal, signifying whether the joint lines of both knees, when extended to the midline, are below, level with, or above the level of a horizontal joint line, respectively, and is calculated as MPTA + LDFA. By combining the three subgroups of αHKA with the three subgroups of JLO, nine CPAK types are derived [63]. Indeed, the αHKA in the CPAK classification system does not take into account the JLCA and is not influenced by joint space narrowing or tibiofemoral subluxation. The system assumes that when the distal femoral and proximal tibial joint lines are parallel, the αHKA is equivalent to the mechanical HKA [5]. Last, because CPAK is limited to two-dimensional evaluation, it does not encompass axial or sagittal alignment, which are also important factors to knee balance.

Another major limitation is the fact that not all the nine possible CPAK phenotypes are represented in the patient population. This is further demonstrated by a newly proposed simple modified CPAK (mCPAK) system, where patients are grouped into 9 mCPAK categories according to whether the femur and tibia are varus, valgus, or neutral: boundaries for neutral are 0° ± 0.5° (Table 2).

**Table 2:** Modified Coronal Plane Alignment of the Knee classification (mCPAK)

	Femur (LDFA)			
Tibia (MPTA)		Valgus	Neutral (0° ± 0.5°)	Varus
	Varus	I Valgus Neutral Varus	II Only Varus	III Only Varus
	Neutral (0° ± 0.5°)	IV Only Valgus	V Neutral	VI Only Varus
	Valgus	VII Only Valgus	VIII Only Valgus	IX Varus Neutral Valgus

Modified Coronal Plane Alignment of the Knee classification (mCPAK) with nine theoretical coronal knee phenotypes and the possible overall coronal alignment type (varus, neutral or valgus) for each one

**Fig. 3:**
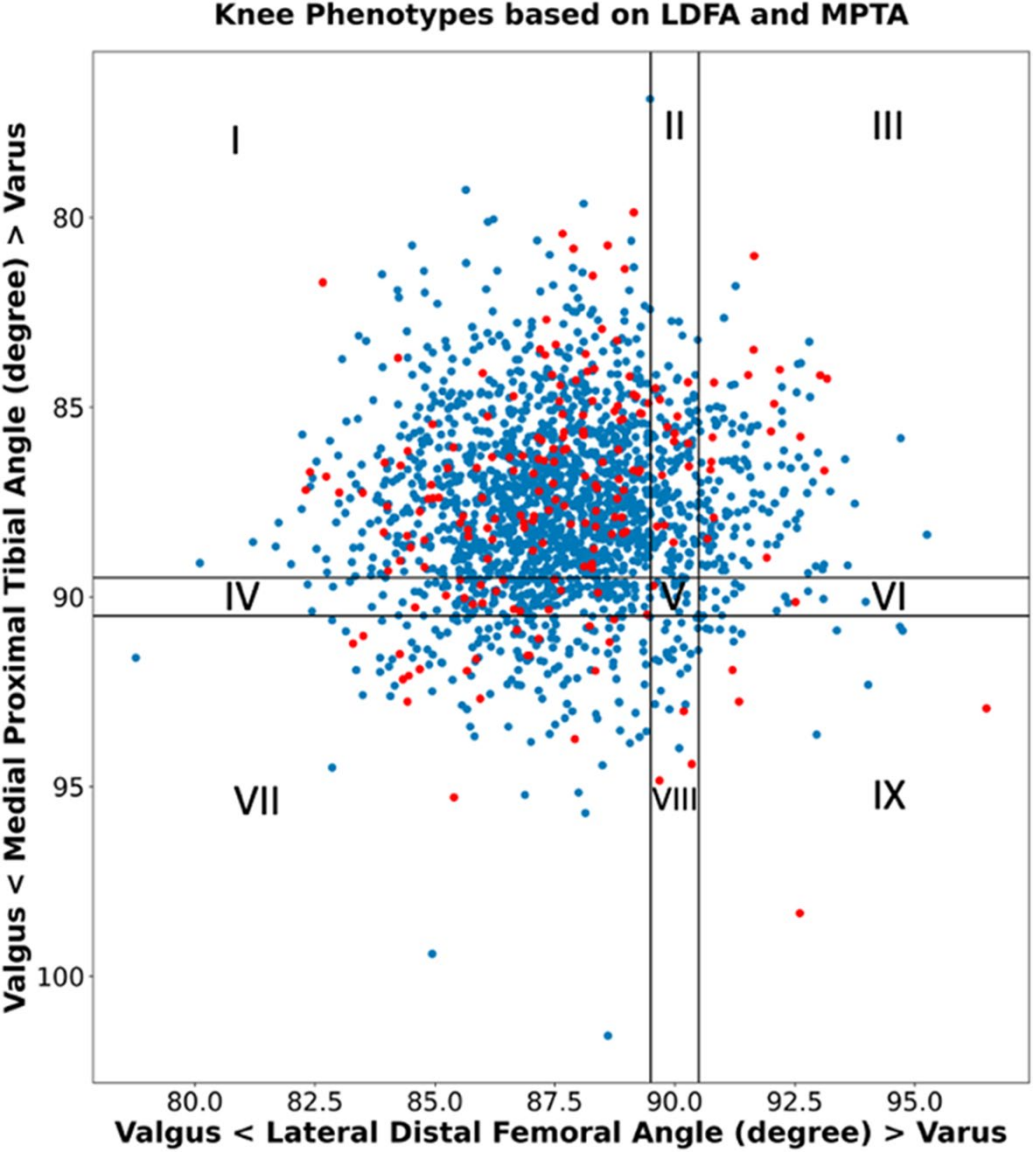
Scatterplot of LDFA against MPTA for 1,944 knees demonstrating distribution by percentage in the mCPAK types, where five types account for 96% of the patients. I: 65.9%, II: 8.6%, III: 8.3%, IV: 6.6%, V: 0.7%, VI: 1.3%, VII: 6.7%, VIII: 0.9%, IX: 0.8% (unpublished data)

Notably, unpublished data of 972 patients (1,944 healthy and arthritic knees) showed that five categories accounted for 96% of the patients (Fig. 3).

The most comprehensive phenotype concept is the functional knee phenotype concept introduced by Hirschmann et al. [18]. There, a knee phenotype (from Greek phanein, meaning “to show” and typos, meaning “type”) is defined as the comprehensive assessment of observable characteristics of the knee, including morphology, alignment, and laxity [31]. Alignment, laxity, and morphology form a functional triad, where one cannot be considered without the other.

First, the system for coronal alignment was presented and it will be followed by sagittal and rotational alignment. In this system, the HKA, FMA, and TMA measurements are conducted medially to ensure coherence. A value above 90° or 180° indicates a varus alignment, while below 90° or 180° signifies a valgus alignment of the femur, tibia, or overall alignment. Phenotype nomenclature consists of three parts for coronal alignment: the first part (NEU, VAR, VAL) indicates the alignment direction, the second subscripted part (HKA, FMA, and TMA) specifies the measured angle, and the last part (0°, 3°, and 6°) indicates the mean deviation of the phenotype from the mean value, with all values falling within a range of ± 1.5° from this mean. Phenotype-specific mean values are represented in 3° increments from the average angle values (HKA: 180°; FMA: 93°; TMA: 87°), with a 3° range chosen first to account for surgical instrument accuracy. Second, by setting a 3° range for the phenotypes, the three central phenotypes (NEU0°, VAR3°, VAL3°) encompass a span equivalent to 1.5–2.5 standard deviations, representing more than 80% to 90% of the population. With five limb, femoral, and tibial phenotypes each, there are theoretically 125 possible combinations. Based on the assessment of CT data from 308 native knees, 43 phenotypes were identified in the non-osteoarthritic population [18]. The functional knee phenotype offers a comprehensive evaluation of an individual’s anatomy and holds significant value in facilitating personalized TKA approaches.

For joint play, gap geometry or laxity of the joint the authors have presented their findings for varus knees yet. Graichen et al. investigated 1000 navigated TKAs of varus knees in various flexion angles, to find out whether all varus knees behave similarly or have more individual soft tissue patterns. Varus OA knees demonstrated large variability regarding their gap widths from extension to flexion with the mean lateral extension gap (4.1 mm) being significantly larger than the medial extension gap (0.6 mm) [24]. These findings suggest that varus knees should not be treated as a uniform entity as they also vary in gap widths at different joint positions. In addition, Mullaji et al. assessed the soft-tissue envelope in 90° of flexion in a consecutive series of valgus arthritic knees and showed that the lateral flexion gap in valgus knees may be narrower than the medial flexion gap, especially in knees with > 10° deformity [64]. This comes in contrast with varus and native knees, in which the lateral flexion gap exceeds the medial gap and suggests that restoring flexion gap balance, may improve outcomes in valgus knees. Analyzing and taking into account the unique envelope of laxity specific to each knee undergoing TKA is essential. Traditionally, all gaps are treated as equal, but a more anatomical approach should be pursued, taking into account individual variations in laxity to achieve optimal outcomes [31]. However, current sensor technology appears to be limited [65].

As third pillar of the functional knee phenotype concept one should consider the individual knee morphology. In particular including the shape of the trochlea, the patella as well as the femoral condyles [66, 67]. Several anthropometrics studies have shown significant variations in tibial geometric ratios among individuals, surpassing the influence of gender and racial differences, making it likely that a substantial portion of knees will experience bone-implant mismatch when surgeons typically employ a single or a limited number of TKA brands [66]. It has also been demonstrated that deviations between the native and prosthetic trochlear sulcus orientations can be substantial, depending on factors such as the native LDFA, implant positioning technique, and the distal trochlear sulcus angle of the implant [68]. At present, we do not have the knowledge of the optimal approach to address each potential anatomic variation, but it is improbable that a one-size-fits-all implant position would be the solution, especially when dealing with more pronounced anatomical differences. Statistical shape modelling and AI technology will help to shed more light into the variability of knee morphology [69–72]. Our understanding of the intricate variability in knee morphology may be enhanced by AI enabled analyses of extensive datasets of knee structures. This could in turn lead to the detection of subtle patterns and associations within the data, aiding in the development of more personalized TKAs, where accommodating individual anatomical variations is crucial for optimizing patient outcomes.

## Conclusion

The concept that a single target alignment approach suits all cases in TKA is being challenged. However, in order to find the correct target, one should first define the individual knee phenotypes via a comprehensive analysis of coronal alignment, as well as sagittal and rotational. In this way, bone cuts can be preplanned and the amount of ligament balancing can be eventually diminished. A potential avenue for improving TKA outcomes lies in the combination of achieving mechanically sound prosthetic alignment while respecting the soft tissue envelope surrounding the knee joint. The high precision offered by enhanced technology enables the attainment of personalized implantation targets in a reproducible manner, holding the potential to significantly increase patient satisfaction. The critical importance of determining the most appropriate alignment strategy for each patient becomes evident, as the magnitude of bone resection varies markedly based on both the patient’s phenotype and the alignment strategy selected. However, implementing individualized alignment strategies requires careful consideration, and future knee studies should focus on reporting alignment, positioning, and balance reproducibly to ensure consistency and reliability in research findings.

## Data Availability

Data sharing is not applicable to this article as no datasets were analyzed during the current study.

## Abbreviations

AA: Anatomic alignment
AMA: Adjusted mechanical alignment
aHKA: Arithmetic hip knee ankle angle
CPAK: Coronal plane alignment of the knee
dMCL: Deep medial collateral ligament
ER: External rotation
FA: Functional alignment
FMA: Femoral mechanical angle
HKA: Hip knee ankle angle
iKA: Inverse kinematic alignment
JLO: Joint line obliquity
JLCA: Joint line convergence angle
KA: Kinematic alignment
LDFA: Lateral distal femoral angle
MA: Mechanical alignment
mCPAK: Modified coronal plane alignment of the knee
MPTA: Medial proximal tibial angle
rKA: Restricted kinematic alignment
TKA: Total knee arthroplasty
TMA: Tibial mechanical angle

## References

[1] Font-Rodriguez DE, Scuderi GR, Insall JN (1997). Survivorship of cemented total knee arthroplasty. Clin Orthop Relat Res.

[2] Rodricks DJ, Patil S, Pulido P, Colwell CW (2007). Press-fit condylar design total knee arthroplasty. Fourteen to seventeen-year follow-up. J Bone Joint Surg Am.

[3] DeFrance MJ, Scuderi GR (2023). Are 20% of patients actually dissatisfied following total knee arthroplasty? A systematic review of the literature. J Arthroplasty.

[4] Mathis DT, Hirschmann MT (2021). Why do knees after total knee arthroplasty fail in different parts of the world?. J Orthop.

[5] Karasavvidis T, Pagan Moldenhauer CA, Haddad FS, Hirschmann MT, Pagnano MW, Vigdorchik JM (2023). Current concepts in alignment in total knee arthroplasty. J Arthroplasty.

[6] Almaawi AM, Hutt JRB, Masse V, Lavigne M, Vendittoli P-A (2017). The impact of mechanical and restricted kinematic alignment on knee anatomy in total knee arthroplasty. J Arthroplasty.

[7] Bellemans J, Colyn W, Vandenneucker H, Victor J (2012). The Chitranjan Ranawat award: is neutral mechanical alignment normal for all patients? The concept of constitutional varus. Clin Orthop Relat Res.

[8] Sappey-Marinier E, Shatrov J, Batailler C, Schmidt A, Servien E, Marchetti E, Lustig S (2022). Restricted kinematic alignment may be associated with increased risk of aseptic loosening for posterior-stabilized TKA: a case-control study. Knee Surg Sports Traumatol Arthrosc.

[9] Oussedik S, Abdel MP, Victor J, Pagnano MW, Haddad FS (2020). Alignment in total knee arthroplasty. Bone Joint J.

[10] Begum FA, Kayani B, Magan AA, Chang JS, Haddad FS (2021). Current concepts in total knee arthroplasty : mechanical, kinematic, anatomical, and functional alignment. Bone Jt Open.

[11] Hood B, Blum L, Holcombe SA, Wang SC, Urquhart AG, Goulet JA, Maratt JD (2017). Variation in optimal sagittal alignment of the femoral component in total knee arthroplasty. Orthopedics.

[12] Berend ME, Ritter MA, Meding JB, Faris PM, Keating EM, Redelman R, Faris GW, Davis KE (2004). Tibial component failure mechanisms in total knee arthroplasty. Clin Orthop Relat Res.

[13] Fang DM, Ritter MA, Davis KE (2009). Coronal alignment in total knee arthroplasty: just how important is it?. J Arthroplasty.

[14] Nam D, Nunley RM, Barrack RL (2014). Patient dissatisfaction following total knee replacement: a growing concern?. Bone Joint J.

[15] Ritter MA, Davis KE, Meding JB, Pierson JL, Berend ME, Malinzak RA (2011). The effect of alignment and BMI on failure of total knee replacement. J Bone Joint Surg Am.

[16] Werner FW, Ayers DC, Maletsky LP, Rullkoetter PJ (2005). The effect of valgus/varus malalignment on load distribution in total knee replacements. J Biomech.

[17] Rivière C, Lazic S, Boughton O, Wiart Y, Vïllet L, Cobb J (2018). Current concepts for aligning knee implants: patient-specific or systematic?. EFORT Open Rev.

[18] Hirschmann MT, Moser LB, Amsler F, Behrend H, Leclerq V, Hess S (2019). Functional knee phenotypes: a novel classification for phenotyping the coronal lower limb alignment based on the native alignment in young non-osteoarthritic patients. Knee Surg Sports Traumatol Arthrosc.

[19] Hess S, Moser LB, Amsler F, Behrend H, Hirschmann MT (2019). Highly variable coronal tibial and femoral alignment in osteoarthritic knees: a systematic review. Knee Surg Sports Traumatol Arthrosc.

[20] Hess S, Moser LB, Robertson EL, Behrend H, Amsler F, Iordache E, Leclercq V, Hirschmann MT (2022). Osteoarthritic and non-osteoarthritic patients show comparable coronal knee joint line orientations in a cross-sectional study based on 3D reconstructed CT images. Knee Surg Sports Traumatol Arthrosc.

[21] Hirschmann MT, Moser LB, Amsler F, Behrend H, Leclercq V, Hess S (2019). Phenotyping the knee in young non-osteoarthritic knees shows a wide distribution of femoral and tibial coronal alignment. Knee Surg Sports Traumatol Arthrosc.

[22] Jenny J-Y, Baldairon F, Hirschmann MT (2022). Functional knee phenotypes of OA patients undergoing total knee arthroplasty are significantly more varus or valgus than in a non-OA control group. Knee Surg Sports Traumatol Arthrosc.

[23] Moser LB, Hess S, Amsler F, Behrend H, Hirschmann MT (2019). Native non-osteoarthritic knees have a highly variable coronal alignment: a systematic review. Knee Surg Sports Traumatol Arthrosc.

[24] Graichen H, Lekkreusuwan K, Eller K, Grau T, Hirschmann MT, Scior W (2022). A single type of varus knee does not exist: morphotyping and gap analysis in varus OA. Knee Surg Sports Traumatol Arthrosc.

[25] Hirschmann MT, Hess S, Behrend H, Amsler F, Leclercq V, Moser LB (2019). Phenotyping of hip-knee-ankle angle in young non-osteoarthritic knees provides better understanding of native alignment variability. Knee Surg Sports Traumatol Arthrosc.

[26] Schelker BL, Moret CS, von Eisenhart-Rothe R, Graichen H, Arnold MP, Leclercq V, Huegli RW, Hirschmann MT (2023). The impact of different alignment strategies on bone cuts for neutral knee phenotypes in total knee arthroplasty. Knee Surg Sports Traumatol Arthrosc.

[27] Schelker BL, Moret CS, Sava MP, von Eisenhart-Rothe R, Graichen H, Arnold MP, Leclercq V, Hirschmann MT (2023). The impact of different alignment strategies on bone cuts in total knee arthroplasty for varus knee phenotypes. Knee Surg Sports Traumatol Arthrosc.

[28] Insall JN, Binazzi R, Soudry M, Mestriner LA (1985). Total knee arthroplasty. Clin Orthop Relat Res.

[29] Manjunath KS, Gopalakrishna KG, Vineeth G (2015). Evaluation of alignment in total knee arthroplasty: a prospective study. Eur J Orthop Surg Traumatol.

[30] Rivière C, Iranpour F, Auvinet E, Aframian A, Asare K, Harris S, Cobb J, Parratte S (2017). Mechanical alignment technique for TKA: are there intrinsic technical limitations?. Orthop Traumatol Surg Res.

[31] von Eisenhart-Rothe R, Lustig S, Graichen H, Koch PP, Becker R, Mullaji A, Hirschmann MT (2022). A safe transition to a more personalized alignment in total knee arthroplasty: the importance of a “safe zone” concept. Knee Surg Sports Traumatol Arthrosc.

[32] Schelker BL, Nowakowski AM, Hirschmann MT (2022). What is the “safe zone” for transition of coronal alignment from systematic to a more personalised one in total knee arthroplasty? A systematic review. Knee Surg Sports Traumatol Arthrosc.

[33] Luderer V, Strauch M, Hirschmann MT, Graichen H (2023). Independent of the preoperative coronal deformity, adjusted mechanical alignment leads in a high percentage to non-anatomical tibial and femoral bone cuts. Knee Surg Sports Traumatol Arthrosc.

[34] Hommel H, Perka C, Pfitzner T (2016). Preliminary results of a new surgical technique in total knee arthroplasty (TKA) using the native ligament tension for femoral implant positioning in varus osteoarthritis. Arch Orthop Trauma Surg.

[35] Hommel H, Tsamassiotis S, Falk R, Fennema P (2020). Adjusted mechanical alignment: operative technique-Tips and tricks. Orthopade.

[36] Graichen H, Luderer V, Strauch M, Hirschmann MT, Scior W (2023). Navigated, gap-balanced, adjusted mechanical alignment achieves alignment and balancing goals in a very high percentage but with partially non-anatomical resections. Knee Surg Sports Traumatol Arthrosc.

[37] Howell SM, Shelton TJ, Hull ML (2018). Implant survival and function ten years after kinematically aligned total knee arthroplasty. J Arthroplasty.

[38] Lustig S, Sappey-Marinier E, Fary C, Servien E, Parratte S, Batailler C (2021). Personalized alignment in total knee arthroplasty: current concepts. SICOT J.

[39] Rivière C, Lazic S, Villet L, Wiart Y, Allwood SM, Cobb J (2018). Kinematic alignment technique for total hip and knee arthroplasty: the personalized implant positioning surgery. EFORT Open Rev.

[40] Hungerford DS, Krackow KA (1985). Total joint arthroplasty of the knee. Clin Orthop Relat Res.

[41] Klatt BA, Goyal N, Austin MS, Hozack WJ (2008). Custom-fit total knee arthroplasty (OtisKnee) results in malalignment. J Arthroplasty.

[42] Cherian JJ, Kapadia BH, Banerjee S, Jauregui JJ, Issa K, Mont MA (2014). Mechanical, anatomical, and kinematic axis in TKA: concepts and practical applications. Curr Rev Musculoskelet Med.

[43] Howell SM, Howell SJ, Kuznik KT, Cohen J, Hull ML (2013). Does a kinematically aligned total knee arthroplasty restore function without failure regardless of alignment category?. Clin Orthop Relat Res.

[44] Winnock de Grave P, Kellens J, Tampere T, Vermue H, Luyckx T, Claeys K (2023). Clinical outcomes in TKA are enhanced by both robotic assistance and patient specific alignment: a comparative trial in 120 patients. Arch Orthop Trauma Surg.

[45] Elbuluk AM, Jerabek SA, Suhardi VJ, Sculco PK, Ast MP, Vigdorchik JM (2022). Head-to-head comparison of kinematic alignment versus mechanical alignment for total knee arthroplasty. J Arthroplasty.

[46] Lung BE, Donnelly MR, McLellan M, Callan K, Amirhekmat A, McMaster WC, Yang S, So DH (2023). Kinematic alignment may reduce opioid consumption and length of stay compared to mechanically aligned total knee arthroplasty. Orthop Surg.

[47] Shatrov J, Coulin B, Batailler C, Servien E, Walter B, Lustig S (2023). Alignment philosophy influences trochlea recreation in total knee arthroplasty: a comparative study using image-based robotic technology. Int Orthop.

[48] Hutt JRB, LeBlanc M-A, Massé V, Lavigne M, Vendittoli P-A (2016). Kinematic TKA using navigation: surgical technique and initial results. Orthop Traumatol Surg Res.

[49] Laende EK, Richardson CG, Dunbar MJ (2019). A randomized controlled trial of tibial component migration with kinematic alignment using patient-specific instrumentation versus mechanical alignment using computer-assisted surgery in total knee arthroplasty. Bone Joint J.

[50] Waterson HB, Clement ND, Eyres KS, Mandalia VI, Toms AD (2016). The early outcome of kinematic versus mechanical alignment in total knee arthroplasty: a prospective randomised control trial. Bone Joint J.

[51] Young SW, Walker ML, Bayan A, Briant-Evans T, Pavlou P, Farrington B (2017). The Chitranjan S. Ranawat Award: no difference in 2-year functional outcomes using kinematic versus mechanical alignment in TKA: a randomized controlled clinical trial. Clin Orthop Relat Res.

[52] Vendittoli P-A, Martinov S, Blakeney WG (2021). Restricted kinematic alignment, the fundamentals, and clinical applications. Front Surg.

[53] Winnock de Grave P, Kellens J, Luyckx T, Tampere T, Lacaze F, Claeys K (2022). Inverse kinematic alignment for total knee arthroplasty. Orthop Traumatol Surg Res.

[54] Shatrov J, Battelier C, Sappey-Marinier E, Gunst S, Servien E, Lustig S (2022). Functional Alignment Philosophy in Total Knee Arthroplasty - rationale and technique for the varus morphotype using a CT based robotic platform and individualized planning. SICOT J.

[55] Shatrov J, Foissey C, Kafelov M, Batailler C, Gunst S, Servien E, Lustig S (2023). Functional Alignment Philosophy in Total Knee Arthroplasty-rationale and technique for the valgus morphotype using an image based robotic platform and individualized planning. J Pers Med.

[56] Blakeney W, Clément J, Desmeules F, Hagemeister N, Rivière C, Vendittoli P-A (2019). Kinematic alignment in total knee arthroplasty better reproduces normal gait than mechanical alignment. Knee Surg Sports Traumatol Arthrosc.

[57] Choi BS, Kim SE, Yang M, Ro DH, Han H-S (2023). Functional alignment with robotic-arm assisted total knee arthroplasty demonstrated better patient-reported outcomes than mechanical alignment with manual total knee arthroplasty. Knee Surg Sports Traumatol Arthrosc.

[58] Hovinga KR, Lerner AL (2009). Anatomic variations between Japanese and Caucasian populations in the healthy young adult knee joint. J Orthop Res.

[59] Moser LB, Hess S, de Villeneuve Bargemon J-B, Faizan A, LiArno S, Amsler F, Hirschmann MT, Ollivier M (2022). Ethnical differences in knee phenotypes indicate the need for a more individualized approach in knee arthroplasty: a comparison of 80 Asian knees with 308 Caucasian knees. J Pers Med.

[60] Pagan CA, Karasavvidis T, Lebrun DG, Jang SJ, MacDessi SJ, Vigdorchik JM (2023). Geographic variation in knee phenotypes based on the coronal plane alignment of the knee classification: a systematic review. J Arthroplasty.

[61] Cooke D, Scudamore A, Li J, Wyss U, Bryant T, Costigan P (1997). Axial lower-limb alignment: comparison of knee geometry in normal volunteers and osteoarthritis patients. Osteoarthritis Cartilage.

[62] Lin Y-H, Chang F-S, Chen K-H, Huang K-C, Su K-C (2018). Mismatch between femur and tibia coronal alignment in the knee joint: classification of five lower limb types according to femoral and tibial mechanical alignment. BMC Musculoskelet Disord.

[63] MacDessi SJ, Griffiths-Jones W, Harris IA, Bellemans J, Chen DB (2021). Coronal Plane Alignment of the Knee (CPAK) classification. Bone Joint J.

[64] Mullaji A, Singh A, Haidermota M (2022). Arthritic knees with more than 10° valgus can have soft-tissue imbalance in flexion. Knee Surg Sports Traumatol Arthrosc.

[65] Sava M-P, Hara H, Alexandra L, Hügli RW, Hirschmann MT (2023). Verasense sensor-assisted total knee arthroplasty showed no difference in range of motion, reoperation rate or functional outcomes when compared to manually balanced total knee arthroplasty: a systematic review. Knee Surg Sports Traumatol Arthrosc.

[66] Beckers L, Müller JH, Daxhelet J, Ratano S, Saffarini M, Aït-Si-Selmi T, Bonnin MP (2023). Considerable inter-individual variability of tibial geometric ratios renders bone-implant mismatch unavoidable using off-the-shelf total knee arthroplasty: a systematic review and meta-analysis. Knee Surg Sports Traumatol Arthrosc.

[67] Beckers L, Müller JH, Daxhelet J, Saffarini M, Aït-Si-Selmi T, Bonnin MP (2022). Sexual dimorphism and racial diversity render bone-implant mismatch inevitable after off-the-shelf total knee arthroplasty: a systematic review and meta-analysis. Knee Surg Sports Traumatol Arthrosc.

[68] Borukhov I, Esposito CI, Ismailidis P, LiArno S, Lyon JP, McCarthy TF, McEwen P (2023). The trochlear sulcus of the native knee is consistently orientated close to the sagittal plane despite variation in distal condylar anatomy. Knee Surg Sports Traumatol Arthrosc.

[69] Dahmen J, Kayaalp ME, Winkler PW, Ollivier M, Pareek A, Karlsson J, Hirschmann MT (2023). Intelligent innovations for our journal’s path forward. Knee Surg Sports Traumatol Arthrosc.

[70] Dobbelaere A, Müller JH, Aït-Si-Selmi T, Gousopoulos L, Saffarini M, Bonnin MP (2023). Sagittal femoral condylar shape varies along a continuum from spherical to ovoid: a systematic review and meta-analysis. Arch Orthop Trauma Surg.

[71] Hinterwimmer F, Lazic I, Langer S, Suren C, Charitou F, Hirschmann MT, Matziolis G, Seidl F, Pohlig F, Rueckert D, Burgkart R, von Eisenhart-Rothe R (2023). Prediction of complications and surgery duration in primary TKA with high accuracy using machine learning with arthroplasty-specific data. Knee Surg Sports Traumatol Arthrosc.

[72] Hinterwimmer F, Lazic I, Suren C, Hirschmann MT, Pohlig F, Rueckert D, Burgkart R, von Eisenhart-Rothe R (2022). Machine learning in knee arthroplasty: specific data are key-a systematic review. Knee Surg Sports Traumatol Arthrosc.

